# 2491. The Association of Parkinson’s Disease with Hepatitis C Virus Infection and Its Treatment

**DOI:** 10.1093/ofid/ofad500.2109

**Published:** 2023-11-27

**Authors:** Samia Aslam, Peng Yan, Adeel A Butt

**Affiliations:** VA Pittsburgh Healthcare System, Pittsburgh, PA; VA Pittsburgh Healthcare System, Pittsburgh, PA; VA Pittsburgh Healthcare System, Pittsburgh, PA

## Abstract

**Background:**

There are contradicting reports regarding the relationship between hepatitis C virus (HCV) infection and its treatment with idiopathic Parkinson’s Disease (PD). We sought to determine the association of HCV infection and its treatment with idiopathic PD.

**Methods:**

We used the Electronically Retrieved Cohort of HCV Infected Veterans (ERCHIVES), as well characterized national cohort of HCV infected Veterans and uninfected controls to create a dataset of HCV infected individuals and propensity-score matched (PSM) uninfected controls, matched on age, race, sex, body mass index, using nearest neighbor matching with a caliper of 0.2 SD. We excluded individuals with prevalent PD diagnosis (any PD diagnosis before the first HCV positive test date and the match date for corresponding controls), HIV or hepatitis B virus coinfection from both groups, and those with missing or undetectable baseline HCV RNA values among the HCV infected group. PD was defined as presence of > 1 inpatient or > 2 outpatient ICD-10 CM codes. HCV treatment was defined as receipt of any FDA-approved treatment with combination directly acting antiviral agents for > 30 consecutive days. Cox regression analysis was used to determine the probability of disease-free survival for HCV infected and uninfected, and among the HCV infected for those treated and untreated for HCV.

**Results:**

We identified a total of 25,579 matched pairs. Incident idiopathic PD was diagnosed in 344 (1.34%) of HCV+ and 252 (0.99%) of HCV- individuals. Among the HCV+, 9,445 (36.9%) were treated and 91 (0.96%) were diagnosed with PD; while 16,134 were untreated and 161 (1.00%) were diagnosed with PD. Kaplan-Meier curves showed no difference in the probability of remaining PD-free between HCV+ and HCV- (log-rank p=0.5; **see figure**), while among the HCV+, those who were treated had a higher probability of remaining PD-free over median of 10.5 years (IQR 5.3-15.6; maximum 23.6 years) of follow-up (log-rank p < 0.001; **see figure**).

**Figure:**
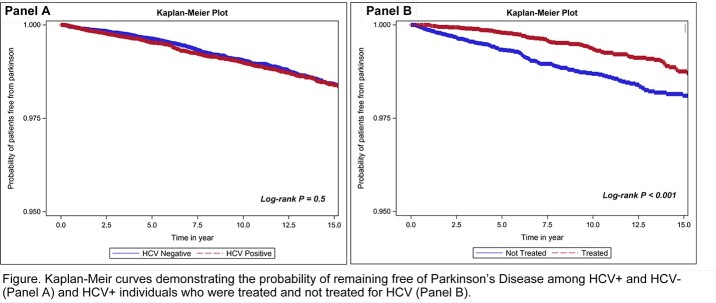

**Conclusion:**

While we did not find a difference incidence of idiopathic PD among HCV+ vs. HCV- individuals, HCV+ individuals who received anti-HCV treatment were less likely to develop incident idiopathic PD over a median of 10.5 years of follow up.

**Disclosures:**

**Adeel A. Butt, MBBS, MS**, Gilead Sciences: Grant/Research Support|Merck and Company: Grant/Research Support

